# Heterogeneous slab thermal dehydration driving warm subduction zone earthquakes

**DOI:** 10.1038/s41598-023-48498-3

**Published:** 2023-11-30

**Authors:** Ye Zhu, Yingfeng Ji, Lijun Liu, Weiling Zhu, Rui Qu, Chaodi Xie, Haris Faheem, Shoichi Yoshioka, Lin Ding

**Affiliations:** 1 State Key Laboratory of Tibetan Plateau Earth System, Environment and Resources (TPESER), Institute of Tibetan Plateau Research, Chinese Academy of Sciences, Beijing, 100101 China; 2 University of Chinese Academy of Sciences, Beijing, 100049 China; 3 State Key Laboratory of Lithospheric Evolution, Institute of Geology and Geophysics, Chinese Academy of Sciences, Beijing, 100029 China; 4 University of Illinois at Urbana-Champaign, Urbana, IL USA; 5 Geophysics Department, School of Earth Sciences, Yunnan University, Kunming, 650500 China; 6 Research Center for Urban Safety and Security, Kobe University, Kobe, Japan; 7 Department of Planetology, Graduate School of Science, Kobe University, Kobe, Japan

**Keywords:** Geophysics, Seismology, Geodynamics

## Abstract

Changing thermal regime is one of the key mechanisms driving seismogenic behaviors at cold megathrusts, but it is difficult to interpret warm subduction zones such as Vanuatu for the temperatures are higher than that accommodates shallow brittle failures. We construct a 3-D thermomechanical model to clarify the thermal structure that controls tectonic seismicity in Vanuatu and predict a warm circumstance associated with abundant seismicity. Results reveal a heterogeneous slab ranging from 300 °C to over 900 °C from the Moho to subvolcanic depth. The subduction seismicity corresponds well to the plate interface where dynamic thermal dehydration is focused. The transformation from hydrated basalts to eclogites along the slab facilitates the occurrence of intense earthquakes and slips. Multistage mineralogical metamorphism affects the dynamic stability of megathrusts and favors the generation of active interplate large events. Therefore, slab thermal dehydration plays a greater role than slab temperature condition in influencing the subduction earthquake distribution in warm subduction systems.

## Introduction

**Figure 1:**
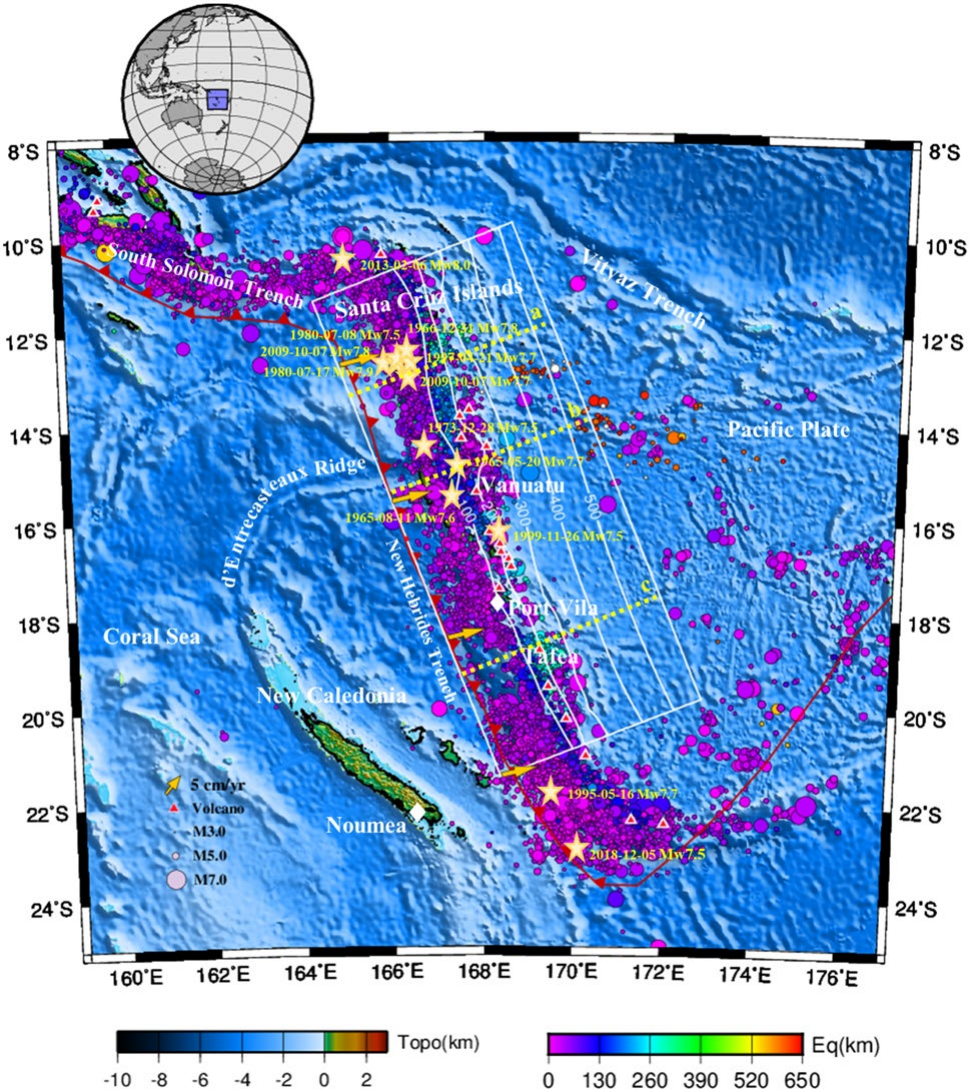
Tectonic map. The background color, shown by the “Topo” color scale in the bottom left corner of the figure, with zero being sea level, indicates the surface topography (ETOPO1-Bedrock)^76^. The white line indicates the study region and the isodepth contours on the subducted Pacific Plate, with a contour interval of 100 km (Slab2)^31^. The yellow arrows show the convergence velocity. The red barbs on the red line mark the convergent plate boundary. The red triangles with white outlines indicate volcanoes^77^. The solid circles indicate earthquakes of all magnitudes that occurred from January 2000 through December 2010 (IRIS)^74^ and M > 5.5 earthquakes that occurred from January 1900 through December 2000 (Centennial)^78^. Earthquake magnitudes are indicated by the circle sizes, as shown by the legend in the bottom right corner of the map. The five-point stars with labels indicate M ≥ 7.5 earthquakes that have occurred in the past century. The three dashed yellow lines a-c indicate the profiles of the three cross-sections (Fig. S3) in northern, central, and southern Vanuatu, respectively.

**Figure 2:**
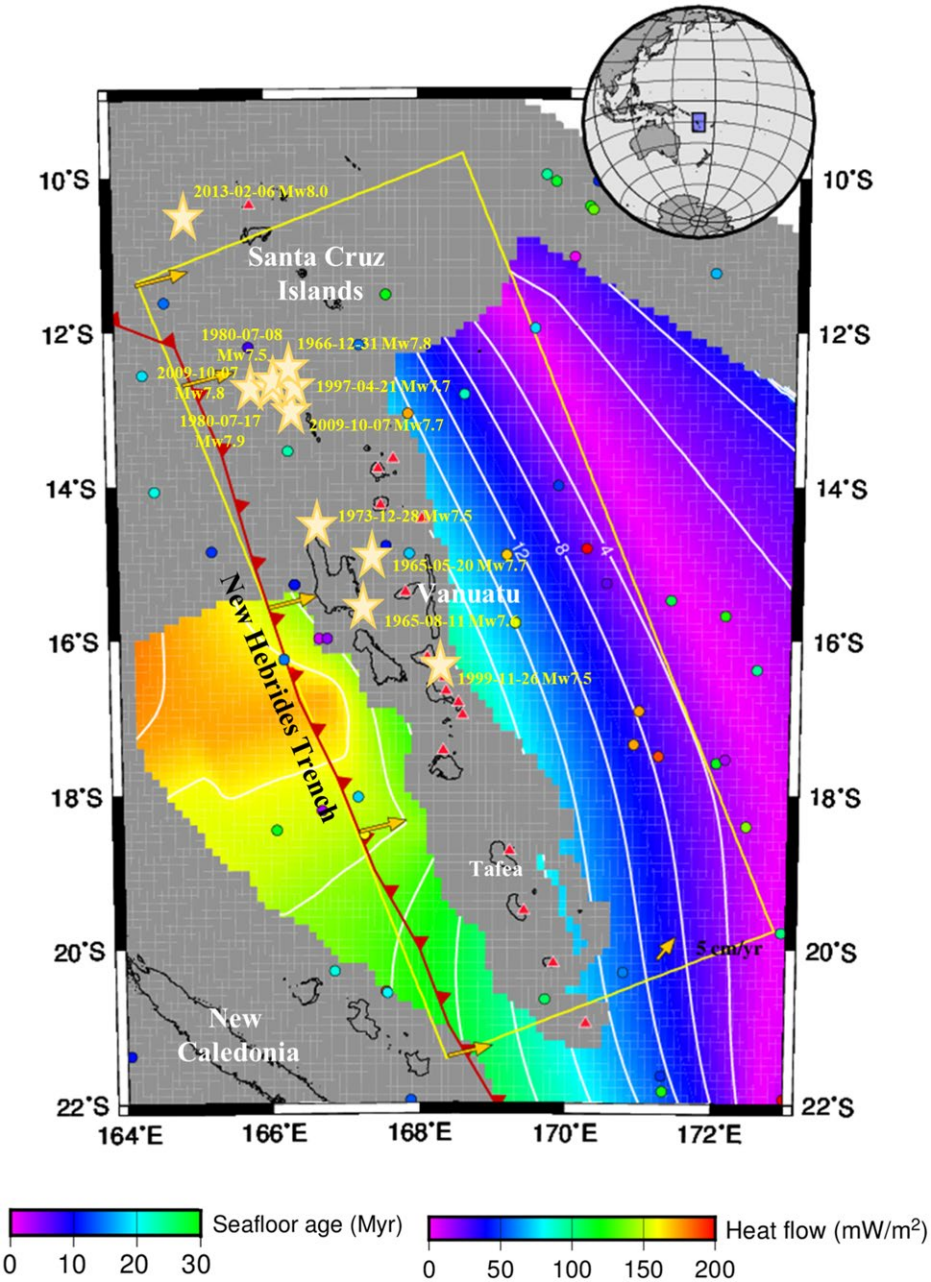
Seafloor age and heat flow in Vanuatu. The yellow line indicates the study region. The yellow arrows show the convergence velocity. The red barbs on the black solid line mark the convergent plate boundary. The red triangles with white outlines indicate volcanoes^77^. The solid circles represent observations from the global heat flow database, with the circle color corresponding to the “Heat flow” color scale in the bottom right corner of the figure^36^. The seafloor age, with the color corresponding to the “Seafloor age” color scale in the bottom left corner of the figure, was obtained from EarthByte^33^. The gold stars indicate M ≥ 7.5 earthquakes that have occurred in the past century.

Subduction zones are the primary study areas for dynamic, thermal and petrologic evolutional processes, where the downgoing crust and mantle exchange materials, creating various significant geohazards, including the occurrence of periodic megathrust earthquakes, intense volcanic magmatism, and trenchward-oriented tsunamis^1^. Subducted plate age is considered to contribute to the subduction thermal structure as well as the faulting behavior types^2^. In old and cold subduction zones, temperatures in the forearc need to be cold enough to allow brittle, stick–slip behavior along the megathrust (≤ 450 °C)^3–5^, while in the case of young and warm subduction zones (e.g., Vanuatu, Cascadia, southwest Japan, and Ecuador), the plate interface temperature is estimated to be 200 ~ 300 °C higher than that of cold subduction zones at a depth of 15 ~ 60 km^5^ according to the P‒T of exhumed rocks^6,7^. This increases the urgency of reevaluating the thermal structure of warm subduction zones. Great destructive thrust earthquakes are a characteristic of many warm subduction zones, such as southwest Japan, Cascadia, and Costa Rica, where megathrust earthquakes have occurred repeatedly, e.g., the M8 Tonankai (1944) and Nankai (1946), M9 Cascadia (1700), and M7.6 Costa Rica (2012) earthquakes. In southwest Japan, oblique subduction with a curved geometry played a key role in affecting the intraplate temperature distribution of the Philippine Sea plate and led to a cold anomaly at the plate interface beneath the Bungo Channel and western Shikoku^8^. In these regions, temperatures in the slab core at a depth near the continental Moho were approximately 200 °C lower than those in eastern Shikoku, suggesting high thermal lateral heterogeneity within the subducting plate^8^. In Cascadia, seismicity is confined to shallow depths off Vancouver Island and northern California and projects to greater depths beneath Washington and southern British Columbia^9^. The comparison of seismicity patterns with thermo-petrologic constraints and longwave length slab geometry indicates that the occurrence of seismicity in Cascadia is dominated by an interplay between metamorphic dehydration within the subducting oceanic plate, slab strain, and a plate boundary seal that controls where fluids enter the overriding plate^9^. Vanuatu is also a warm subduction system because the seafloor age along the New Hebrides trench varies from 20 to 50 Myr, and the megathrust features frequent M > 6 earthquakes and slips (Figs. 1 and 2). More than 50 large earthquakes (> Mw7) have occurred in the Vanuatu region since 1900, where an average of one calamitous Mw7 + earthquake has been recorded per year since 1972, including the 1999 Mw7.5^10^, 2013 Mw8.0^11,12^, and 2013 Mw8.0 earthquakes with slow slip^13^.

The convergent boundary between the Australian plate and the Pacific plate corresponds to a broader area of deformation, including the Tonga and Vanuatu (formerly New Hebrides) subduction zones^14,15^. The estimated ages of the oceanic plate being subducted beneath Vanuatu Island range from 13 to 50 Ma, implying a high degree of heterogeneity^16^. The Vanuatu arc (11–22°S, 166–171°E, Fig. 1) in the southwestern region of the Pacific Ocean is an approximately 1200 km-long island arc^17^ that is generated by the interaction and volcanism between the North Fiji basin and Vanuatu plates^18,19^. The New Hebrides trench was generated by the eastward subduction of the Australian plate beneath the New Hebrides arc and the North Fiji Basin (Fig. 1)^20^, which has been significantly modified by its collision with several major submarine ridges and plateaus^21^. The convergence rates are 11.8 ~ 10.3 cm/yr between the Vanuatu arc and the Australian plate (Figs. 1 and 2)^22^. The Australian plate subducts in a nearly trench-orthogonal convergence, where the subduction interface has a shallow dip (< 15°) over the initial 20 ~ 40 km from the New Hebrides trench and then rapidly bends downward, reaching a potential 40° dip at a depth of ~ 40 km (Fig. 1)^1,17^.

**Figure 3:**
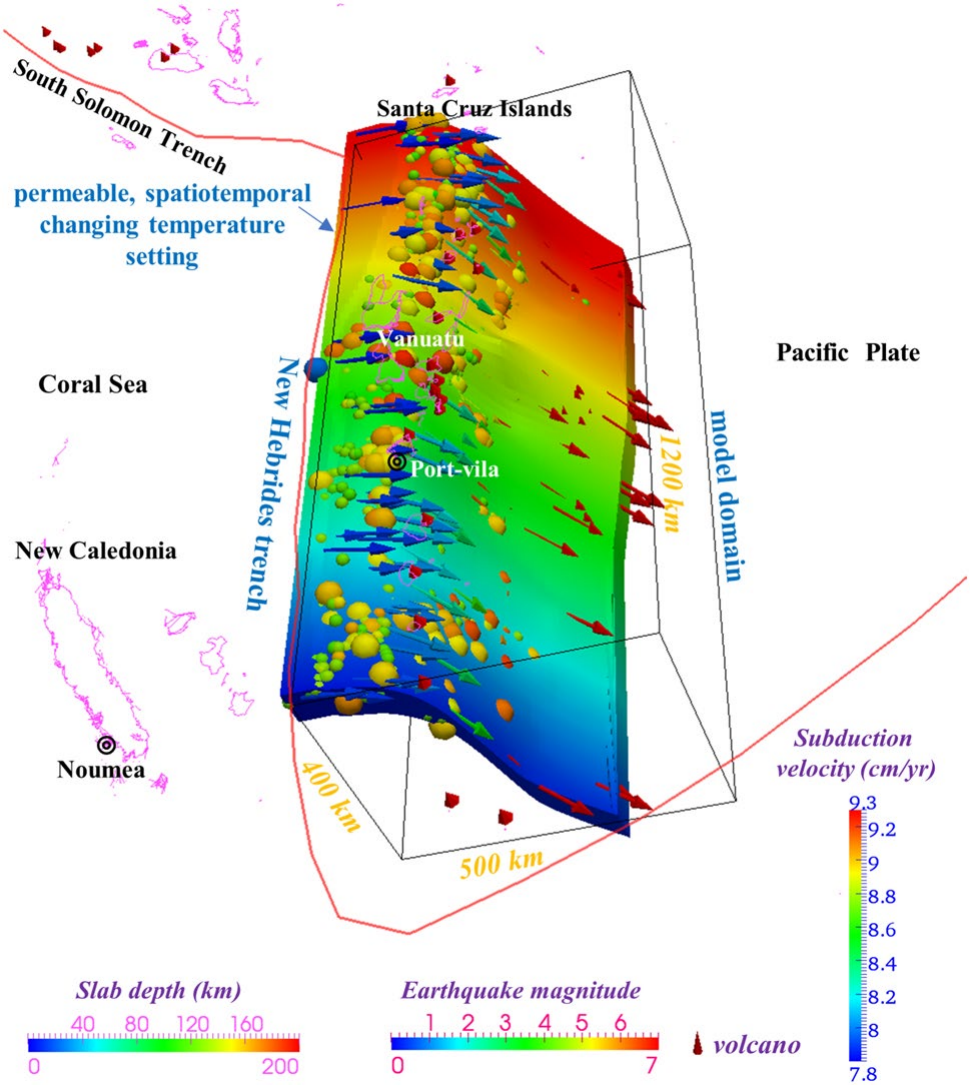
Model setting and boundary conditions. The solid red line is the plate boundary. The slab depth is shown by the “Slab depth” color scale in the bottom left corner of the figure. The arrows indicate the plate subduction velocity, as shown by the vertical “Subduction velocity” color scale in the bottom right corner of the figure. The intraslab seismic events from January 2000 through December 2010 (IRIS)^74^ are represented by colored spheres, with the color indicating the earthquake magnitude, as shown by the “Earthquake magnitude” color scale at the bottom of the figure. The red cones indicate active arc volcanoes^77^.

Preceding studies of the upper mantle and lithographic crust located beneath the Vanuatu arc have revealed complicated subsurface structures and deformation processes that are connected with its subducting features^19,23^. In recent decades, the development of subsurface thermal measurement capabilities has greatly helped delineate the regions of hydrothermal fluid release within areas of intense seismic and volcanic activity^24^. Many studies have indicated that the variations in earthquakes occurring within an intermediate depth range are likely related to the slab dehydration mechanism as well as the depths and temperatures at which dehydration embrittlement occurs^25,26^. However, the thermal regime of the subduction zone associated with tectonic seismic activity on the subducting Pacific Plate in the Vanuatu Arc is poorly understood. In this paper, we employ a 3-D time-dependent thermomechanical model (Fig. 3) to study the thermal regime, petrological metamorphism, and seismicity occurring under the Vanuatu subduction zone and attempt to fill some knowledge gaps based on previously conducted studies on the Vanuatu arc, which may provide a window into the associations between the slab dehydration process and heterogeneous megathrust seismicity.

## Materials and methods

In this study, a combined geodynamic and thermodynamic subduction model allowed the prediction of mineral dehydration and variation in the slab thermal state during the plate subduction process. We constructed a 3-D thermomechanical kinematic model (Fig. 3) that combined the finite difference method (FDM), finite volume method (FVM) and Stag3D code^27^ for the Vanuatu subduction zone, with dimensions of 1200 km × 500 km × 400 km (length × width × depth) and 80 × 80 × 100 grid points. An anelastic liquid approximation and the equations of conservation of mass, momentum, and energy were applied in this model^8,28^. The plate subduction period was set to 20 Myr, which allowed the model to reach steady-state thermal conditions, with a temperature variation of less than 0.1% (< 10 °C) over time and a lapse time of ≥ 5 Myr^29,30^. The geometry of the subducted oceanic plate followed that in Slab 2.0^31^ within the modeled domain to reach a state of full subduction and comparatively steady-state thermal conditions (Figs. 2 and 3). The thickness of the oceanic lithosphere ranges from ~ 70 km to ~ 80 km, and these values were used in the model^32,33^. The seafloor age varies from the northeastern part of Vanuatu (~ 2 Myr) to the central segment of the New Hebrides trench (~ 42 Myr, depicted in Fig. 2). The trenchward temperature boundary was assumed by utilizing the plate cooling model^34^, and the plate ages were assigned by EarthByte^33^. The bottom and vertical model boundary interfaces were set to be adiabatic and permeable, and the top surface interface was stipulated to be at a fixed temperature (set to 0 °C and rigid. The convection velocities inside a set 3-D constrained volume of the oceanic lithosphere were given according to the kinematic plate subduction modeling method^8,35^, with velocities increasing from 78 mm/yr in the southern New Hebrides area to 93 mm/yr in the northern Vanuatu area (shown in Fig. 3). The global surface heat flow database^36^ and the Curie depth estimates^37^ were used to constrain the thermal regime modeling results (Fig. S1).

The model involves 3-D geometric data for the incoming plate updated through seismic tomography (Slab2)^31^ and real subduction velocities from the MORVEL^38,39^ data sets. The subduction velocities inside a prescribed 3-D constrained volume of the oceanic lithosphere are given based on the kinematic plate subduction modeling method^8^:

$${v}_{x}(x,y,z)=\frac{-2a(x,y)b(x,y){v}_{y}+\sqrt{\{2a(x,y)b(x,y){v}_{y}{\}}^{2}-4\{a(x,y{)}^{2}+1\}[\{a(x,y{)}^{2}+1\}{{v}_{y}}^{2}-{v}^{2}]}}{2\{a(x,y{)}^{2}+1\}}$$

$${v}_{y}(x,y,z)={v}_{y},$$

$${v}_{z}(x,y,z)=a(x,y){v}_{x}+b(x,y){v}_{y},$$

While.

$$a(x,y)=\frac{1}{2}\{Z(x+\Delta x,y)-Z(x-\Delta x,y)\}\cdot \frac{{z}_{max}}{{x}_{max}},$$

$$b(x,y)=\frac{1}{2}\{Z(x+\Delta x,y+\Delta y)-Z(x+\Delta x,y)+Z(x-\Delta x,y)-Z(x-\Delta x,y-\Delta y)\}\cdot \frac{{z}_{max}}{{y}_{max}}.$$

Here, v is the subduction velocity, and Δ is the interval between two neighboring nodes along the axes. ${x}_{max}$, ${y}_{max}$, and ${z}_{max}$ indicate the model lengths along the x, y, and z axes, respectively. We performed sensitivity tests to investigate the robustness of our modeling results and varied the mantle viscosity from 1.0 × 10^19^ Pa s to 1.0 × 10^21^ Pa s and the mantle density from 3250 kg/m^3^ to 3350 kg/m^3^. We present the benchmark model results as deviations from the reference models (ΔT and ΔH_2_O) and show these results at different depth levels within the oceanic slab. The tests show that mantle density variations (± 50 kg/m3) induce small temperature variations of < 10 °C at depth.

In our petrological modeling method, existing beneath the thick layer of bottom sediment, MORBs were regarded as the major component of the uppermost part of the oceanic plates^40,41^, and harzburgite was assumed to be the dominant underlying ultramafic rock^25^. We established a P‒T–wt%–facies database based on the methodology outlined previously by Omori et al.^40^ (for MORB) and Hacker et al.^25^, with a P‒T grid interval of 0.04 GPa (1.2 km) and 5 °C. From the calculated 3-D thermal model results, we obtained the temperature at every P‒T grid point. The pressure (GPa) at every grid point was obtained by converting its depths (km) via the parameters of the preliminary reference Earth model (PREM). Based on the P‒T conditions provided by the numerical simulation, we estimated the intraslab water content distribution (wt%) and intraslab dehydration distribution (wt%/km) at various depths through the interpolation calculation method. More detailed model configuration selections, initial boundary conditions and physical parameters are available in the Supplementary Information (SI).

## Results

### 3-D thermal states and temperature gradient variation

**Figure 4:**
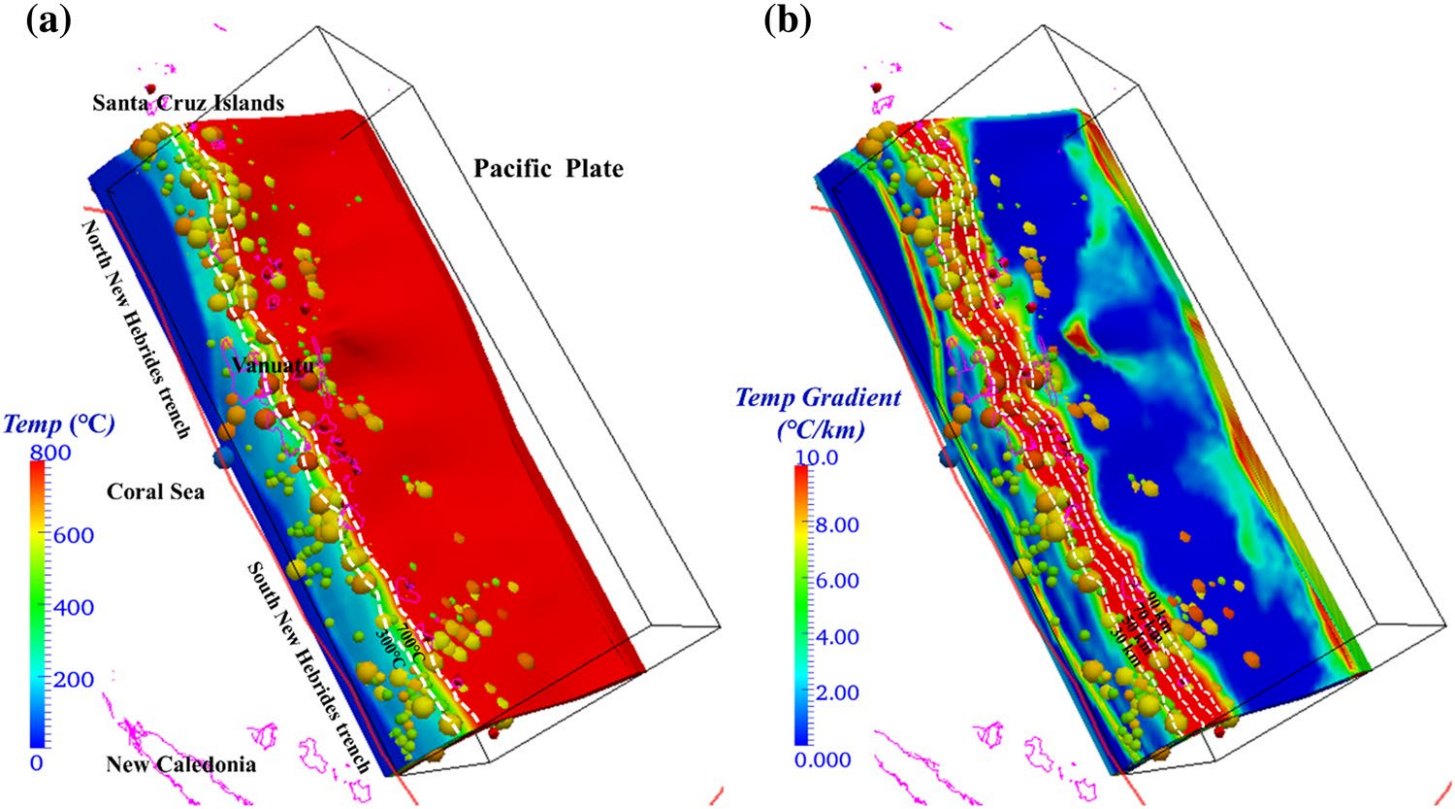
Calculated thermal state of the upper surface of Vanuatu. (**a**) The calculated temperature distribution. The white dashed lines represent the isotherm contours of 300 °C and 700 °C. (**b**) The calculated temperature gradient distribution. The white dashed lines represent the depth contours of 30 km, 50 km, 70 km, and 90 km. In both figure panels, the solid red lines show the plate boundary. The red cones indicate active arc volcanoes^77^. The colored spheres indicate the intraslab seismic events from January 2000 through December 2010 (IRIS)^74^, with the color indicating the earthquake magnitude and the size mimicking the rupture dimension.

Utilizing a 3-D model, we estimated the steady-state thermal conditions of the subducting plate in the Vanuatu arc, including the intraslab temperature structure (Fig. 4a) and temperature gradient (Fig. 4b) along the direction of plate subduction. The temperature structure at various depths (0 km, 8 km, 16 km and 24 km) vertically downward from the plate interface was determined (Fig. S2 in Supplementary Information, SI). The subarc temperature results reveal relatively low temperatures from the slab interface to the Moho depth (< 300 °C while the intraslab temperature ranges from 300 °C to 900 °C between the Moho depth and a depth of ~ 70 km, representing the greatest dehydration segment for mid-ocean ridge basalt (MORB). As the subducting plate depth increases to the subvolcanic (100 km) depth, the temperature rises to > 1200 °C and the subvolcanic (100 km) depth exceeds the prescribed plate thickness (~ 70–80 km). Generally, the temperature distribution with respect to depth indicates variation in the intraslab temperature from less than 300 °C to 900 °C The hypocenters of most regular earthquakes (Fig. 3, colored spheres) occurred at depths of < 200 km, which corresponds with the spatial variation in the slab temperature, indicating a possible influence and control mechanism of the intraplate thermal state on the generation of earthquakes at an intermediate depth (< 300 km).

Compared with the observation of an abrupt increase in the intraslab temperature from 400 °C–600 °C to 800 °C–1100 °C beneath the arc in the plate decoupling model conducted by Wada and Wang (2009)^42^, the interplate temperature predicted by our models in Vanuatu without slab-mantle decoupling gradually increases from 300 °C to 800 °C–1200 °C gradually with increasing depth. Previous studies have suggested a > 200 °C decrease in the mantle wedge temperature from the beginning of the plate subduction process to its arrival at steady-state condition^43^, which is consistent with our observation of a decrease in temperature of 200 ~ 500 °C but in contrast to the 200 ~ 800 °C temperature variation in slab–mantle decoupling and cold forearc models. The P‒T records of globally exhumed blueschists and eclogites have been shown to be hotter than previous models suggest^5^, indicating that this discrepancy means that implementation of a completely cold forearc may result in an underestimation of the intraslab temperature by an average of 200 ~ 300 °C. The slab–mantle decoupling setting in the aforementioned modeling may be a significant factor causing 200 ~ 300 °C decreases in the forearc slab surface temperature at a depth of < 80 km. We suggest that thermal models without slab-mantle decoupling may predict a thermal regime that better fits the observed surface heat flow and seismicity distribution in several subduction zones^44,45^.

The intraslab temperature gradient along the upper surface of the Vanuatu plate was also evaluated (Fig. 4b). There is a relatively high temperature gradient along the strike direction of the New Hebrides trench (4 ~ 8 °C/km), and the temperature gradient in the northern Vanuatu region is > 8 °C/km. The gradient associated with the corresponding plate boundary decreases to < 4 °C/km; however, as the vertical depth increases to the Moho depth, the gradient increases again to > 4 °C/km. The thrust surface beneath the Vanuatu Arc exhibits the highest temperature gradient (< 10 °C/km) within a depth range of 30 (Moho depth)-90 km, and then the gradient continuously decreases to > 6 °C/km from depths of 90 ~ 110 km. The other depth regions beneath the Vanuatu Arc exhibit comparatively low temperature gradients (< 4 °C/km). This aberrant temperature distribution suggests that the Vanuatu subduction plate boundary is in an asymmetric thermal state, which is presumably caused by the rapid oblique subduction between the Australian and Pacific plates^1,39^.

### Water content and slab dehydration with mineralogical metamorphism

**Figure 5:**
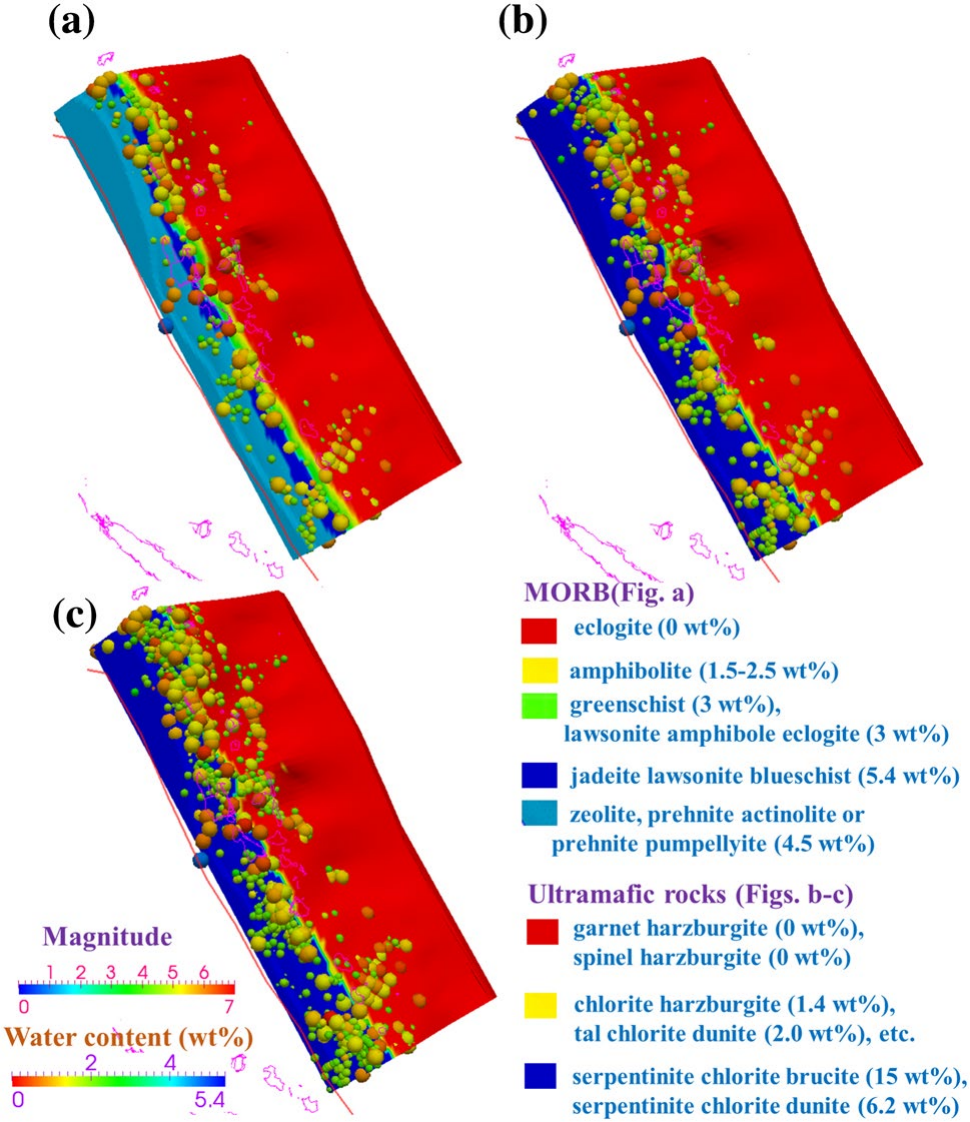
Water content (wt%) of the incoming plate. The solid red lines show the plate boundary. The colored spheres indicate the intraslab seismic events from January 2000 through December 2010 (IRIS)^74^, with the color indicating the earthquake magnitude, as shown by the “Magnitude” color scale in the bottom left corner of the figure. The water content is shown by the “Water content” color scale in the bottom left corner of the figure. The red cones indicate active arc volcanoes^77^. (**a**) The interface. (**b**) 8 km below the interface. (**c**) 16 km below the interface.

In the modeling of the Vanuatu subduction zone, through petrological modeling utilizing the P‒T–wt%–facies database established based on Omori et al.^40^ (for MORB) and Hacker et al.^25^, we calculate the rock facies and corresponding water content (wt%) at every grid point. Then, the difference between the water contents at two adjacent grid points in the subduction direction is divided by the distance between these points, yielding the slab dehydration rate (in wt%/km). The estimated slab hydration state consists of MORBs in the uppermost 7 km-thick layer and ultramafic minerals in the deeper layer, with intraslab temperature variations from 300 °C to 900 °C There is a distinct transition in the intraplate water content at depths of 0 and 16 km below the megathrust (Fig. 5a,c). In the lower oceanic plate region, the water content of the ultramafic minerals is estimated to be ~ 15 wt%, maintaining a steady state until reaching the transition zone (located at a depth of 16 km). As the depth from the subduction interface increases, a large volume of water-rich fluid is released, and the water content decreases to 0 wt%.

**Figure 6:**
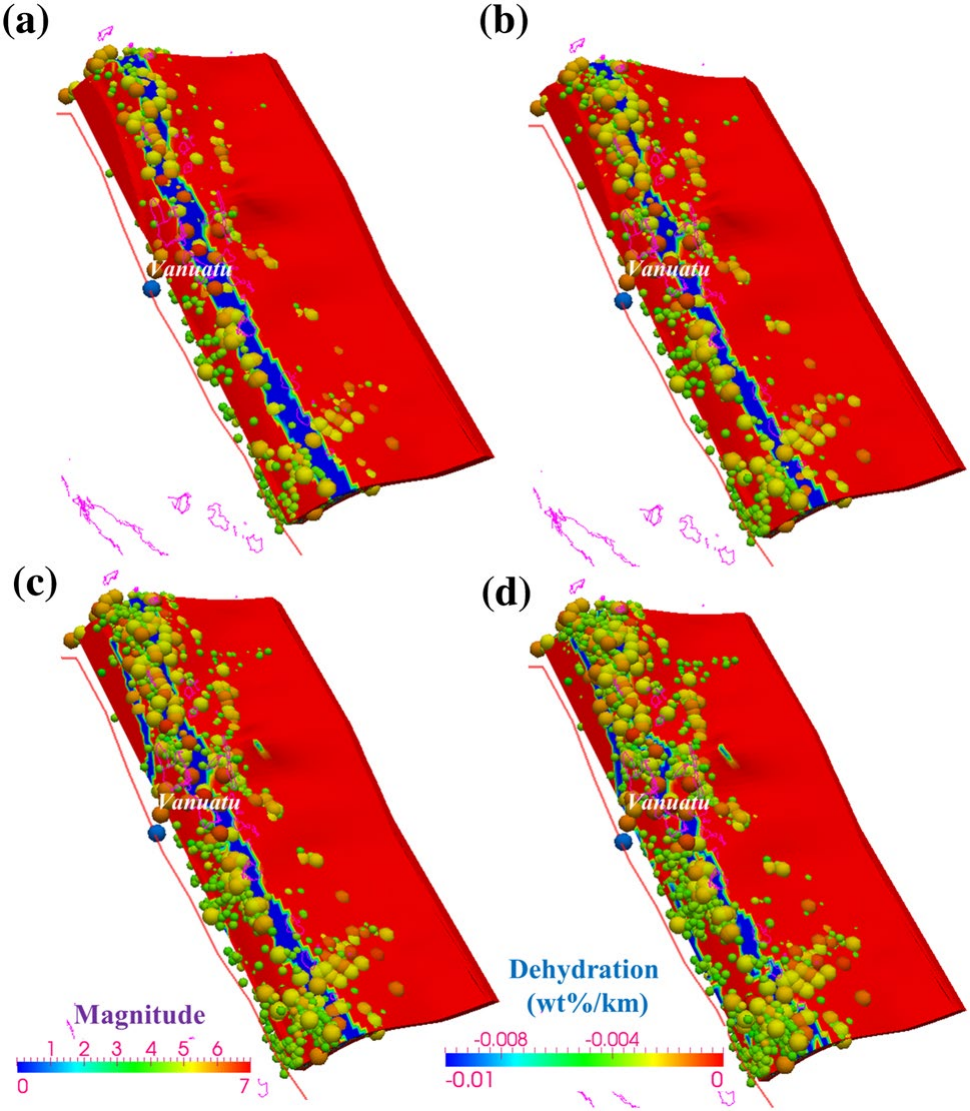
Slab dehydration (wt%/km) of the incoming plate. The solid red lines show the plate boundary. The colored spheres indicate the intraslab seismic events from January 2000 through December 2010 (IRIS)^74^, with the color indicating the earthquake magnitude, as shown by the “Magnitude” color scale at the bottom of the figure. The dehydration is shown by the “Dehydration” color scale in the bottom left corner of the figure. The red cones indicate active arc volcanoes^77^. (**a**) The interface. (**b**) 8 km below the interface. (**c**) 16 km below the interface. (**d**) 24 km below the interface.

In cold subduction zones, major dehydration reactions are predicted to occur at the transition from blueschist- to eclogite-facies conditions (> 60–70 km depth)^46^. In relatively warm subduction zones, consistent with previous studies^47,48^, our results suggest that a range of major dehydration reactions are predicted to occur at shallower depths. Figure 5 presents the complete slab dehydration process accompanied by multistage phase transitions and mineralogical metamorphism, and the maximum rate of slab dehydration is > -0.05 wt%/km for the MORB layer and > − 0.1 wt%/km for the ultramafic layer (Fig. 6). For MORB compositions in the uppermost 7 km-thick layer of the slab, ∼4.5–5.4 wt% H_2_O is bound within zeolite, prehnite actinolite, prehnite pumpellyite, jadeite lawsonite blueschist and other low-grade hydrous minerals at low pressures (< ∼0.7 GPa) and temperatures (< ∼350 °C At higher temperatures (∼450–570 °C mineral-bound water is held in greenschist and lawsonite amphibole eclogite (∼3 wt%) and then amphibolite facies (∼1.5–3 wt%). The MORB is completely dehydrated and transforms into eclogite facies (0 wt%) with higher P–T conditions. In the deeper layer with ultramafic minerals (2 GPa < P < 5 GPa), serpentinite chlorite brucite/dunite is metamorphosed to chlorite dunite/harzburgite and ultimately transformed into garnet/spinel orthopyroxene (0 wt%). Under this P‒T condition, some hot slabs precipitate a large amount of water at T = 600–800 °C (Figs. 4 and 5)^25^, which far exceeds the upper temperature limit of controlling slab brittle failure (450 °C^3–5^. Furthermore, the occurrence of earthquakes relatively corresponds with the predicted locations of plate dehydration fronts present within the subducting slab as it descends, suggesting that slab thermal dehydration plays a greater role than slab temperature conditions in influencing the subduction earthquake distribution.

## Discussion

### The control of thermal states and slab dehydration on seismicity

Vanuatu is an intraoceanic subduction zone with high heat flows and strain rates in the southwest Pacific, where the subduction of the Australian plate at the New Hebrides trench contributes to the increasing slab temperature, the dehydration process and the subsequent release of water-rich fluids, which causes the overlying mantle wedge to melt and produce magmatism^17,49^. Meanwhile, the mantle flow induced by toroidal-type motion brings hotter mantle material from behind the slab into the mantle wedge, elevating the geothermal gradients of the slab edge^2^. Such complex subsurface structures and processes associated with subduction features result in heterogeneous thermal states in the slab, facilitating potential seismic activity. In this process, fluids released from the subducting slab during metamorphic dehydration will allow weak hydrous minerals to form and will elevate pore fluid pressure along the slab-mantle wedge interface. The elevated pore pressure caused by dehydration of the hydrous minerals in the incoming plate can weaken the effective normal stress and thus promote aseismic creeping^25,26^. However, whether the dominant factor controlling the occurrence of earthquakes is slab temperature conditions or dehydration embrittlement during slab mantle devolatilization is ambiguous and worth clarifying.

Evidence for dehydration embrittlement in deformed rocks is abundant in numerous laboratory petrological experiments, including chlorite^50^, serpentine^51^ and amphibolite^52^, which have agreed that even under the P‒T conditions at depths below 70 km where brittle deformation is traditionally considered impossible, rocks could still suffer sudden weakening and brittleness if the permeability is insufficient to release continuously increased fluid pressure during dehydration. Additionally, a test of dehydration of antigorite serpentinite under all conditions (pressures of 1 ~ 6 GPa; temperatures of 650 ~ 820 °C) confirmed that dehydration embrittlement is a viable mechanism for nucleating earthquakes as long as hydrous minerals breakdown under differential stress^53^. The observations of measuring acoustic emission energy during antigorite dehydration in a multianvil press from 1.5 ~ 8.5 GPa to 300 ~ 900 °C revealed brittle deformation features associated with high pore-fluid pressures^51^. Furthermore, within the crust, seismogenesis generally varies with the P–T trajectory^54,55^. In cold subduction zones, earthquakes occur within the crust at pressures of 4–5 GPa (125–160 km depth), some at temperatures of 500–650 °C; however, in warm subduction zones, few earthquakes occur in the crust when the crust passes through these same temperatures, albeit at lower pressures of 0.8–1.5 GPa^56^. Therefore, temperature alone cannot regulate seismogenesis. Moreover, there are clearly temperatures where earthquakes occur in the mantle but not in the crust, so lithology matters much more^56^. This evidence demonstrates that dehydration through different mineral systems potentially plays a more crucial role than temperature conditions in the generation of earthquakes in warm subduction zones.

The USGS earthquake search catalog (Figs. 1, 2 and SI) of the Vanuatu arc reveals shallow (< 70 km) and intermediate-depth (70 ~ 300 km) seismic zones, where the majority of Mw ≥ 7.5 earthquakes have occurred at depths of 10 ~ 55 km since 1960. The focal depths of earthquakes in the slab that exhibit relatively high rates of seismicity are located at < approximately 70 km, with a range of temperature variation of 300 ~ 900 °C (Fig. 4). Fig. S3 shows the cross-sectional profiles of the temperature structure corresponding to lines a, b, and c in Fig. 1, suggesting that the cold mantle wedge (< 500 °C) is constrained at depths of 30 ~ 50 km. Consistent with the studies mentioned in the previous section, our modeling results show that there is no evident control of slab temperature structure on generating earthquakes, while the location of frequent major events clearly corresponds well to a slab dehydration belt (Fig. 6), where the MORB mineral assemblage underwent prehnite actinolite (4.5 wt%)–blueschist (5.4 wt%)–greenschist (3 wt%)–amphibolite (1.5 wt%-2.5 wt%)–eclogite (0 wt%) transitions and serpentinite chlorite brucite/dunite metamorphism from chlorite dunite/harzburgite to garnet/spinel orthopyroxene (0 wt%) (Figs. 5 and S4). These multistage mineralogical metamorphisms are believed to supply abundant fluids to the seismogenic zone below the forearc in warm subduction zones^41,57^, and eclogitization plays a more important role in seismic activity. The dehydration process of serpentinized mantle at temperatures > 600 °C has also been proposed as an explanation for the existence of shallow earthquakes^25^. As suggested for western Chile, this hypothesis requires that the oceanic plate mantle be hydrated down to a depth of 30 km, presumably by deep, Moho-crossing faults before subduction occurs^58^. Fortunately, our modeling results match this hypothesis well, confirming the rationality of the model and indicating that the frequent rate of seismic recurrence (including the recorded Mw > 7.5 earthquakes) is potentially associated with the intraslab high-temperature dehydration process.

On the basis of the International Seismological Centre seismic catalog (which has gathered data since 1910) and GPS results, the presence of a 150 km wide seismic gap with a low seismic P-wave velocity beneath Malekula Island that extends south to Efate Island has been suggested^1,59^. However, interpretation remains debated on the subject of this intermediate-depth seismic gap that has a low seismic P-wave velocity characteristic^17,26^. In accordance with the assumption that seismic gaps are not necessarily associated with the absence of a slab at that location^60^, related investigations have suggested that the observed low seismic P-wave velocities are related to the probable presence of hydrated blueschists^48^ and that the P-wave attenuation extends to deeper depths with the dehydration of these facies^61^. In this paper, our numerical modeling results suggest that the large region at an intermediate depth with reduced seismicity may be caused by reduced slab hydration at that location, and the less recurrence of seismicity could be a sign of efficient slippage resulting from the highly hydrated subducted crust (mantle wedge serpentinization)^19^. These seismic gap low‐velocity anomalies presumably indicate a relatively higher water content in the intensely fractured subducted features through sediments and fluids associated with slab dehydration^19,62^.

### Maximum limit of fast to slow earthquakes

As one of the Earth’s most seismically active regions, the warm subduction zone of Vanuatu has undergone numerous moderate to large earthquakes in recent decades (Fig. 1)^12^. Approximately 20 Mw 5.5 + earthquakes occur in the Vanuatu arc every year, according to the National Earthquake Information Center (NEIC) catalog, and only the Japan and Tonga Trenches have similar earthquake occurrence rates over a long plate boundary distance^63^. More than 50 large earthquakes (> Mw7.0) have occurred in Vanuatu since 1900, and the northern Vanuatu subduction zone (located from 11°S to 14°S) experienced large Mw > 7.5 thrust earthquakes in 1966 (Mw 7.8), 1980 (Mw 7.5 and 7.9), 1997 (Mw 7.7), 2009 (Mw 7.7 and 7.8), and 2013 (Mw 8.0) (18) (Fig. 1). However, despite these abundantly occurring moderate- and large-magnitude earthquakes, this region has not exhibited any events with Mw > 8 since at least 1900^12^, and the cause of this lack of Mw > 8.0 earthquakes (especially at deep depths) remains debated and difficult to determine.

Based on earthquake swarms associated with aseismic slip^64^ and the movement of volatiles in hydrothermal systems^65^, Holtkamp et al.^66^ suggested that the pervasiveness of earthquake swarms along the margin of the Vanuatu arc may indicate the aseismic release of larger moments, thereby preventing the growth of large contiguous ruptures, which may help explain the processes that result in a lack of great M8 + megathrust earthquakes along the margin region. Additionally, several other suggested interpretations for the lack of deep-focus megathrust events have been proposed, including plastic shear^67^ shear instabilities^68^ transformational faulting^69^ and dehydration embrittlement^25,53^. Some investigators have observed that hydroxyl defects precipitate in eclogite at the grain boundaries, which could create a small quantity of melt and generate dehydration-related faulting instability^70^. They believed that this mechanism could be crucial in the mantle transition zone (400–700 km), where earthquakes occur despite the main hydrous phases favoring decomposition in the upper mantle^71^. Theoretically, this instability may lead to synchronous multiple-segment ruptures in Vanuatu and facilitate the future occurrence of earthquakes larger than those recorded to date^12^. Furthermore, in general, the depth zone of the seismogenic layer that facilitates great-magnitude earthquakes is near the mantle wedge corner, which is cooler than the MORB-source mantle^72^ where the dehydrating oceanic crust releases abundant fluid^25^, which is expected to affect the dynamic stability of the megathrust fault and cause large earthquakes to occur in the future by increasing the pore-fluid pressure and/or reducing friction in fault gouges^73^.

Deducing the thermal structure of subduction zones could help our understanding of both the associations between slab dehydration and heterogeneous megathrust seismicity and the process of arc magmatism. In this paper, we construct a 3-D thermomechanical model to investigate petrological metamorphism and seismicity under the Vanuatu subduction zone. The results reveal a heterogeneous slab ranging from 300 °C to over 900 °C from the Moho to subvolcanic depths (Figs. 4, S3). The subduction seismicity corresponds well to the plate interface where dynamic thermal dehydration is focused. The transformation from hydrated basalts to eclogites facilitates the occurrence of intense earthquakes and slips along the slab (Figs. 5, 6 and S4). Multistage mineralogical metamorphism at different temperatures affects the dynamic stability of megathrusts and favors the generation of active interplate large events. Therefore, thermally-controlled slab dehydration plays a greater role than slab temperature conditions in influencing the subduction earthquake distribution in warm subduction systems.

### Supplementary Information

Supplementary Information.

## Data Availability

All data generated or analyzed during this study are included in this published article (and its Supplementary Information files). The data sets generated during and/or analyzed during the current study are available in the Mendeley data repository, https://data.mendeley.com/datasets/kv79vw86jj/1.
