# Supplementary material for: Heterogeneous slab thermal dehydration driving warm subduction zone earthquakes
Source: Sci Rep. 2023 Nov 30;13:21157. doi: 10.1038/s41598-023-48498-3 (PMC10689746; doi:10.1038/s41598-023-48498-3)
Supplement: Supplementary file 1 — Supplementary Information. [file 41598_2023_48498_MOESM1_ESM.pdf]

Supplementary Information for

Heterogeneous slab thermal dehydration driving warm subduction zone earthquakes

Ye Zhu *et al.*

This PDF file includes:

1. Methods and model settings
2. Tables S1-S4
3. Figures S1-S4

Methods and model settings

Based on the thermomechanical model Stag3d (Tackley and Xie, 2003) and the finite difference method (FDM), we investigate the in situ along-strike slab thermal variation in the Vanuatu arc megathrust area and the petrological metamorphic changes in the subducting oceanic lithosphere. An anelastic liquid approximation and the equations of conservation of mass, momentum, and energy are used in this study:

$$\nabla \cdot \{\rho_s(z, T_s)\boldsymbol{v}\} = 0, \tag{1}$$

$$-\frac{\partial P}{\partial x_i} + \frac{\partial \tau_{ij}}{\partial x_j} - \delta_{i3}\rho_s g \alpha_0 (T - T_s) = 0, \tag{2}$$

$$\rho c_p\left(\frac{\partial T}{\partial t} + \boldsymbol{v} \cdot \nabla T\right) = k\nabla^2 T + \eta(\nabla v)^2 + \rho g \alpha T v_z + \rho H_r, \tag{3}$$

where P is the pressure deviation from the hydrostatic pressure, α_0 is the reference thermal expansivity, τ_{ij} (i, j=1, 2, 3) is the stress tensor, and δ_{ij} is the Kronecker delta. The energy equation includes the advection term, thermal diffusion term, viscous dissipation term, adiabatic heating term, and radioactive heating term. The main model parameters are listed in Tables S3 and S4.

The viscous flow law for wet olivine (Burkett and Billen, 2010) following laboratory experiments is included in this study. The deformation of olivine occurs by both diffusion creep (df) and dislocation creep (ds), where each mechanism accommodates a portion of the total strain rate (Hirth and Kohlstedt, 2003):

$$\dot{\varepsilon}_t = \dot{\varepsilon}_{df} + \dot{\varepsilon}_{ds}. \tag{4}$$

The composite upper mantle viscosity under a given condition is as follows:

$$\eta_{comp} = \frac{\eta_{df}\eta_{ds}}{\eta_{df} + \eta_{ds}}, \tag{5}$$

where ηdf and ηds are the diffusion creep and dislocation creep viscosities for olivine, respectively. The general viscosity law is as follows:

$$\eta_{df,ds} = \left(\frac{d^p}{A_0 C_{OH}^r}\right)^{\frac{1}{n}} \dot{\varepsilon}_E^{\frac{1-n}{n}} exp\left(\frac{E_0+P_l V_0}{n_0 R T_a}\right). \quad (6)$$

Here, $\dot{\varepsilon}_E = (\frac{1}{2}\dot{\varepsilon}_{ij}\dot{\varepsilon}_{ij})^{\frac{1}{2}}$ is the square root of the second invariant of the strain rate tensor (e.g., Ranalli, 1995), Ta is the absolute temperature, R is the gas constant, and Pl is the lithostatic pressure. The values of the model parameters for the diffusion and dislocation creep of olivine are tabulated in Table S1. The trenchward thermal structure follows the Global Depth and Heat (GDH1) model (Stein and Stein, 1992; Grose and Afonso, 2013):

$$T(z, t_{oc}) = T_m \left[\frac{z}{d_0} + \frac{2}{\pi}\sum_{n-1}^{\infty}\frac{1}{n}\sin\left(\frac{n\pi z}{d_0}\right)\exp\left(\frac{-n^2\pi^2\kappa t_{oc}}{{d_0}^2}\right)\right] \quad (7)$$

The time-dependent thermal boundary condition includes the initialization time. $T(z, t_{oc})$ is the temperature at depth z and plate age t_{oc} along the trench, Tm is the lithospheric basal temperature, κ is the thermal diffusivity, and d0 is the minimum depth of the adiabatic heating layer.

The model incorporates 3-D geometric data for the incoming plate updated through seismic tomography (Slab2, Hayes et al., 2018) and real subduction velocities from the MORVEL (Argus et al., 2011; DeMets et al., 2010) data sets. The age of the oceanic plate is based on the oceanic seafloor age according to EarthByte (Müller et al., 2008) estimated at the trenchward model boundary. The thickness of the subducting plate is estimated according to the plate age (Yoshii, 1975). The incoming oceanic plate comprises a MORB layer at the top with a thickness of 7 km underlain by an ultramafic rock layer (Hacker et al., 2003). The model dimensions are 1600 km×800 km×400 km (along-arc length×across-arc length×depth) and 80×80×100 grids. The temperature boundary condition agrees with the plate cooling model (Grose and Afonoso, 2013). The bottom of the slab and the vertical planes are prescribed as adiabatic and permeable, and the top surface is set to be a fixed temperature (0°C) and rigid. The subduction velocities inside a prescribed 3-D constrained volume of the oceanic lithosphere are given based on the kinematic plate subduction modeling method (Ji et al., 2016; Ji et al., 2017c):

$$v_x(x,y,z) = \frac{-2a(x,y)b(x,y)v_y + \sqrt{\{2a(x,y)b(x,y)v_y\}^2 - 4\{a(x,y)^2+1\}[\{a(x,y)^2+1\}v_y{}^2 - v^2]}}{2\{a(x,y)^2+1\}},$$

(8)

$$v_y(x,y,z) = v_y, \quad (9)$$

$$v_z(x,y,z) = a(x,y)v_x + b(x,y)v_y, \quad (10)$$

while

$$a(x,y) = \frac{1}{2}[Z(x+\Delta x, y) - Z(x-\Delta x, y)] \cdot \frac{z_{max}}{x_{max}}, \quad (11)$$

$$b(x,y) = \frac{1}{2}[Z(x+\Delta x, y+\Delta y) - Z(x+\Delta x, y) + Z(x-\Delta x, y) - Z(x-\Delta x, y-\Delta y)] \cdot \frac{z_{max}}{y_{max}}. \quad (12)$$

Here, v is the subduction velocity, and Δ is the interval between two neighboring nodes along the axes. x_{max}, y_{max}, and z_{max} indicate the model lengths along the x, y, and z axes, respectively.

Surface heat flow observations from the Global Heat Flow Database (Pollack et al., 1993) and heat flow values from Curie point depth estimates (Li et al., 2017) are used to constrain the model (Fig. S1). Our model follows the trench temperature boundary of the plate cooling model (Stein and Stein, 1992; Grose and Afonso, 2013). We specified the subduction time to be at least 20 Myr to ensure that the model reaches a steady thermal state with a temperature variation <10°C over time with a lapse time of ≥ 5 Myr. We tested the resolution and found that the temperature variance was <1% with a maximum temperature variance <1.9% between 80 × 80 × 100 and 96 × 96 × 100 meshes. We performed sensitivity tests to investigate the robustness of our modeling results and varied the mantle viscosity from 1.0×10^{19} Pa s to 1.0×10^{21} Pa s and the mantle density from 3250 kg/m^3 to 3350 kg/m^3. We present the benchmark model results as deviations from the reference models (ΔT and ΔH_2O) and show these results at different depth levels within the oceanic slab. The tests show that mantle density variations (±50 kg/m3) induce small temperature variations of <10°C at depth.

Ultramafic mantle rocks such as harzburgite (olivine + orthopyroxene) represent the dominant rock type in mantle wedges and the uppermost oceanic mantle, and depleted lherzolite (olivine + orthopyroxene + clinopyroxene) is considered subordinate (Hacker et al., 2003). Seismological studies support the hypothesis that harzburgite represents the principal rock type in the upper mantle. The observed P-wave speeds from White et al. (1992) for the oceanic lower crust and mantle compared with P-wave speeds for various rocks at 200 MPa (Hacker et al., 2003) indicate that most oceanic uppermost mantle (suboceanic mantle) velocity measurements are best explained in terms of spinel harzburgite mantle composition. Due to the reasons above, in our petrological modeling approach, harzburgite is assumed to be the dominant ultramafic rock.

We established a P–T–wt%–facies database according to Omori et al. (2009) (MORB) and Hacker et al. (2003) with a P–T grid interval of 0.04 GPa (1.2 km) and 5°C. The temperature and pressure at every P–T grid point were calculated from our 3D thermal model. The pressure (GPa) at every grid point was obtained by converting its depths (km) through the preliminary reference Earth model (PREM) parameters. Using the temperature and pressure provided by the numerical simulation, we estimated each facies domain and the corresponding water content (wt%) at every grid. Through the interpolation method, we obtained the intraslab water content distribution (wt%) at various depths.

To calculate the intraslab slab dehydration rate, the result of the 3-D water content at every grid was utilized. Slab dehydration (wt%/km) indicates the change in rock saturation water content (wt%) via a distance (km) in the subduction direction between neighboring grids. We prescribed the slab to be divided into >70 layers according to the mesh number, with the layer surface parallel to the plate interface. Then, the water content at a point on a layer surface was calculated from the water content at the grids surrounding this point using an interpolation method. Thus, the water content at each point on every layer surface was obtained. Next, the difference in the water content value between two neighboring points in the subduction direction was divided by the

point distance (which can be derived using the horizontal distance and vertical distance), and then the slab dehydration at each point on a layer could be obtained. Based on these steps, the intraslab dehydration distribution was calculated well. Slab minerals vary in saturation at each subduction stage. Thus, slab dehydration (wt%/km) reflects the efficiency of fluid production by crystalline breakdown and fluid relaxation during subduction along the slab geometry.

The surface heat flow and Curie depth are correlated, following a theoretical thermal conduction relationship (e.g., Li et al., 2011, 2013):

$$Q_s = K\frac{T_c - T_0}{Z_b - Z_s} + h_r^2 H_0 \frac{e^{\frac{-Z_b}{h_r}} - e^{\frac{-Z_s}{h_r}}}{Z_b - Z_s} + h_r H_0 e^{\frac{-Z_s}{h_r}}, \quad (13)$$

where Q_s is the surface heat flow, T_c is the Curie temperature at the Curie depth Z_b, T_0 is the temperature at the surface elevation Z_s, K is the average thermal conductivity of the magnetic layer (Table S4), H_0 is the heat production rate at the surface, and h_r is the characteristic drop-off of heat production. The equation shows a nonlinear inverse relationship between heat flow and Z_b. For the oceanic lithosphere, we assume $H_0 = 1.37$ μW/m^3, $h_r = 5.0$ km, $T_c = 550$ °C, $T_0 = 5$ °C, and $Z_s = 4$ km. For continents, we take $H_0 = 2.0$ μW/m^3, $h_r = 10.0$ km, and $Z_s = -1$ km to account for the larger radioactive contribution (Li et al., 2017). High heat flow measurements tend to be correlated with small Curie depths, and vice versa. The continental data can be best fitted with the theoretical curve of an average thermal conductivity K of ~2.5 W/m°C, and most oceanic data are best fitted with an average K = ~2.0 W/m°C (Li et al., 2013). These conductivities are compatible with those of granite and basalt (e.g., Turcotte and Schubert, 2002). Synthetic modeling suggests that the largest error in the estimated Curie depths using the linearized centroid method does not reach 35%, and the uncertainty of the surface heat flow is expected to be <20 mW/m^2 due to the selected fractal exponent and wavenumber bands for linear regressions and the observed surface heat flow in plate convergence zones (e.g., Li et al., 2013, 2017).

The model settings combine effective simulation methods used in active convergence zones, such as reconstructions of oceanic-continental cold subduction in

eastern Japan (Ji et al., 2017b, 2017c), Hikurangi (Suenaga et al., 2018), and Sumatra (Ji et al., 2021) and warm subduction in southwestern Japan (Ji et al., 2016; Ji and Yoshioka, 2017), Ryukyu (Suenaga et al., 2021), and Cascadia (Ji et al., 2017a). These studies on 3-D thermomechanical models are focused on subducting plates with topographically changing dip angles.

Table S1. Recorded M>7.5 earthquakes in the modeled region (USGS[1], 1960-2020)

time	latitude	longitude	depth (km)	mag	mag_type	net	depthError	magError
2018-12-05T04:18:08.420Z	-21.9496	169.4266	10	7.5	mww	us	1.5	0.047
2013-02-06T01:12:25.830Z	-10.799	165.114	24	8	mww	us		
2009-10-07T22:18:51.240Z	-12.517	166.382	35	7.8	mwc	us		
2009-10-07T22:03:14.470Z	-13.006	166.51	45	7.7	mww	us		
1999-11-26T13:21:15.570Z	-16.423	168.214	33	7.5	mwc	us		
1997-04-21T12:02:26.430Z	-12.584	166.676	33	7.7	mwc	us		
1995-05-16T20:12:44.220Z	-23.008	169.9	20.2	7.7	mw	us		
1980-07-17T19:42:23.200Z	-12.525	165.916	33	7.9	ms	us		
1980-07-08T23:19:19.800Z	-12.41	166.381	33	7.5	ms	us		
1973-12-28T13:41:45.800Z	-14.464	166.601	26	7.5	ms	us		
1966-12-31T18:23:09.430Z	-12.091	166.552	55	7.8	mw	iscgem	5.4	0.4
1965-08-11T22:31:51.450Z	-15.861	167.092	30	7.6	mw	iscgem	4.7	0.3
1965-05-20T00:40:26.460Z	-14.805	167.495	120	7.7	mw	iscgem	13.1	0.4

[1] The earthquake catalog was downloaded from the USGS website (https://earthquake.usgs.gov/earthquakes/search/). These earthquakes are labeled by the five-pointed stars in Figs. 1-2.

Table S2. Recorded M>7.0 earthquakes in the modeled region (USGS[1], 1960-2020)

time	latitude	longitude	depth (km)	mag	mag_type	net	depthError	magError
2018-12-05T04:18:08.420Z	-21.9496	169.4266	10	7.5	mww	us	1.5	0.047
2018-08-29T03:51:56.100Z	-22.0295	170.1262	21.43	7.1	mww	us	2.8	0.049
2015-10-20T21:52:02.560Z	-14.8595	167.3028	135	7.1	mww	us	1.8	
2013-02-08T15:26:38.470Z	-10.928	166.018	21	7.1	mww	us		
2013-02-06T01:23:19.760Z	-11.183	164.882	10	7.1	mww	us		
2013-02-06T01:12:25.830Z	-10.799	165.114	24	8	mww	us		
2012-02-02T13:34:40.650Z	-17.827	167.133	23	7.1	mww	us		
2011-08-20T18:19:23.550Z	-18.311	168.218	28	7.1	mww	us		
2011-08-20T16:55:02.810Z	-18.365	168.143	32	7.2	mww	us		
2010-12-25T13:16:37.000Z	-19.702	167.947	16	7.3	mwc	us		
2010-08-10T05:23:44.980Z	-17.541	168.069	25	7.3	mwc	us		
2010-05-27T17:14:46.570Z	-13.698	166.643	31	7.2	mwc	us		
2009-10-07T23:13:48.160Z	-13.093	166.497	31.1	7.4	mwc	us	2.4	
2009-10-07T22:18:51.240Z	-12.517	166.382	35	7.8	mwc	us		
2009-10-07T22:03:14.470Z	-13.006	166.51	45	7.7	mww	us		
2008-04-09T12:46:12.720Z	-20.071	168.892	33	7.3	mwc	us		
2007-09-02T01:05:18.150Z	-11.61	165.762	35	7.2	mwc	us		
2007-08-01T17:08:51.400Z	-15.595	167.68	120	7.2	mwb	us		
2007-03-25T00:40:01.610Z	-20.617	169.357	34	7.1	mwb	us		
2004-01-03T16:23:21.020Z	-22.253	169.683	22	7.1	mwc	us		
2003-12-27T16:00:59.450Z	-22.015	169.766	10	7.3	mwc	us		
2002-01-02T17:22:48.760Z	-17.6	167.856	21	7.2	mwc	us		
2001-01-09T16:49:28.000Z	-14.928	167.17	103	7.1	mwb	us		
1999-11-26T13:21:15.570Z	-16.423	168.214	33	7.5	mwc	us		

[1] The earthquake catalog was downloaded from the USGS website (https://earthquake.usgs.gov/earthquakes/search/).

time	latitude	longitude	depth (km)	mag	mag_type	net	depthError	magError
1999-02-06T21:47:59.470Z	-12.853	166.697	90.1	7.3	mwc	us		
1998-01-04T06:11:58.970Z	-22.301	170.911	100.6	7.5	mwc	us		
1997-04-21T12:02:26.430Z	-12.584	166.676	33	7.7	mwc	us		
1995-05-16T20:12:44.220Z	-23.008	169.9	20.2	7.7	mw	us		
1994-07-13T02:35:56.020Z	-16.62	167.518	33	7.2	mw	us		
1993-03-06T03:05:49.870Z	-10.972	164.181	20.4	7.1	mwb	us		
1992-10-11T19:24:26.290Z	-19.247	168.948	129	7.4	mw	us		
1990-08-12T21:25:21.950Z	-19.435	169.132	140.4	7.1	mw	us		
1990-07-27T12:37:59.550Z	-15.355	167.464	125.7	7.2	mw	us		
1990-03-05T16:38:12.570Z	-18.318	168.063	20.7	7.1	mw	us		
1986-01-15T20:17:42.710Z	-21.277	170.102	150	7.1	mw	us		
1985-12-21T01:13:22.410Z	-13.966	166.516	43	7.1	mw	us		
1985-11-28T02:25:42.340Z	-14.043	166.24	33	7	mw	us		
1984-11-15T02:46:19.880Z	-22.022	170.95	104.7	7.1	mw	us		
1980-10-25T11:00:05.100Z	-21.89	169.853	33	7.2	ms	us		
1980-07-17T19:42:23.200Z	-12.525	165.916	33	7.9	ms	us		
1980-07-08T23:19:19.800Z	-12.41	166.381	33	7.5	ms	us		
1974-01-10T08:51:13.300Z	-14.434	166.863	34	7.2	ms	us		
1973-12-29T00:19:31.100Z	-15.117	166.896	47	7.2	ms	us		
1973-12-28T13:41:45.800Z	-14.464	166.601	26	7.5	ms	us		
1972-02-14T23:29:53.170Z	-11.481	166.339	101.8	7.4	mw	iscgem	5.2	0.4
1972-01-23T21:17:54.540Z	-13.248	166.407	35	7.05	mw	iscgem	17.8	0.35
1971-11-21T05:57:12.850Z	-11.857	166.557	113.1	7.1	mw	iscgem	5.3	0.3
1969-01-19T18:50:53.820Z	-14.849	167.345	112.9	7.1	mw	iscgem	5.3	0.3
1966-12-31T22:15:19.180Z	-12.326	166.491	35	7.13	mw	iscgem	24.6	0.2
1966-12-31T18:23:09.430Z	-12.091	166.552	55	7.8	mw	iscgem	5.4	0.4

[1] The earthquake catalog was downloaded from the USGS website (https://earthquake.usgs.gov/earthquakes/search/).

time	latitude	longitude	depth (km)[2]	mag	mag_type	net	depthError	magError
1965-08-13T12:40:33.440Z	-16.022	166.97	25	7.4	mw	iscgem	12.2	0.3
1965-08-11T22:31:51.450Z	-15.861	167.092	30	7.6	mw	iscgem	4.7	0.3
1965-08-11T03:40:58.570Z	-15.449	166.98	25	7.2	mw	iscgem	4.5	0.3
1965-05-20T00:40:26.460Z	-14.805	167.495	120	7.7	mw	iscgem	13.1	0.4
1964-07-09T16:39:51.600Z	-15.534	167.666	130	7.16	mw	iscgem	5.2	0.3
1963-09-17T19:20:14.140Z	-10.466	165.36	45	7.21	mw	iscgem	5.3	0.2
1963-09-15T00:46:55.110Z	-10.522	165.642	35	7.4	mw	iscgem	5.1	0.4
1961-07-23T21:51:09.750Z	-18.307	168.29	25	7.33	mw	iscgem	4.5	0.31

[1] The earthquake catalog was downloaded from the USGS website (https://earthquake.usgs.gov/earthquakes/search/).

Table S3. Main model parameters

	Model parameters	Value	Units
ρ_0	Standard density	3300[a]	$kg\cdot m^{-3}$
α_0	Standard thermal expansion	3×10^{-5}[b]	K^{-1}
T_0	Standard temperature	1600	K
k_0	Standard thermal conductivity	2.9[c]	$W\cdot m^{-1}\cdot K^{-1}$
Hr	Radioactive heat generation rate in mantle	2.245×10^{-13}[a]	$W\cdot m^{-3}$
C_{p_0}	Standard specific heat at constant pressure	1200[a]	$J\cdot kg^{-1}\cdot K^{-1}$
κ_0	Standard thermal diffusivity	7.6×10^{-7}[d]	$m^2\cdot s^{-1}$
η_0	Standard viscosity	1×10^{20}[c]	$Pa\cdot s$
v	Subduction velocity	7.8~9.3[e]	$cm\cdot y^{-1}$

		Diffusion creep[f]	Dislocation creep[f]
n_0	Stress exponent	1.0	3.5
A_0	Preexponential factor	1.0	9.0×10^{-20}
C_{OH}	OH concentration ($H/10^6$ Si)	1000	1000
r	C_{OH} exponent	1.0	1.2
E_0	Activation energy (kJ/mol)	335	480
V_0	Activation volume (m^3/mol)		
	Upper mantle	4.0×10^{-6}	11.0×10^{-6}
	Lower mantle	1.5×10^{-6}	-
d	Grain size (μm)		
	Upper mantle	10,000	-
	Lower mantle	40,000	-

[a] Wang et al. (1995) [b] Iwamori (1997) [c] Christensen (1996) [d] Yoshioka and Murakami (2007)
[e] Argus et al. (2011) [f] Hirth and Kohlstedt (2003)

Table S4 Parameters for model domains

Domains	Upper crust	Lower crust	Slab	Mantle	Accretionary prism
Density (kg/m^3)	2600	2900	3300	3300	2600
Viscosity (Pa·s)	1×10^{20}	1×10^{20}	1×10^{20}	1×10^{20}	1×10^{20}
Radioactive heat (W/m^3)	7.3×10^{-10}	1.4×10^{-10}	2.245×10^{-13}	2.245×10^{-13}	7.3×10^{-10}
for magnetic layers:					
Oceanic	1.37×10^{-6}				
Continental	2.0×10^{-6}				
Thermal conductivity (W/m·K)	2.5	2.5	2.5	2.5	1.4
for magnetic layers:					
Oceanic	2.0				
Continental	2.5				

Fig. S1

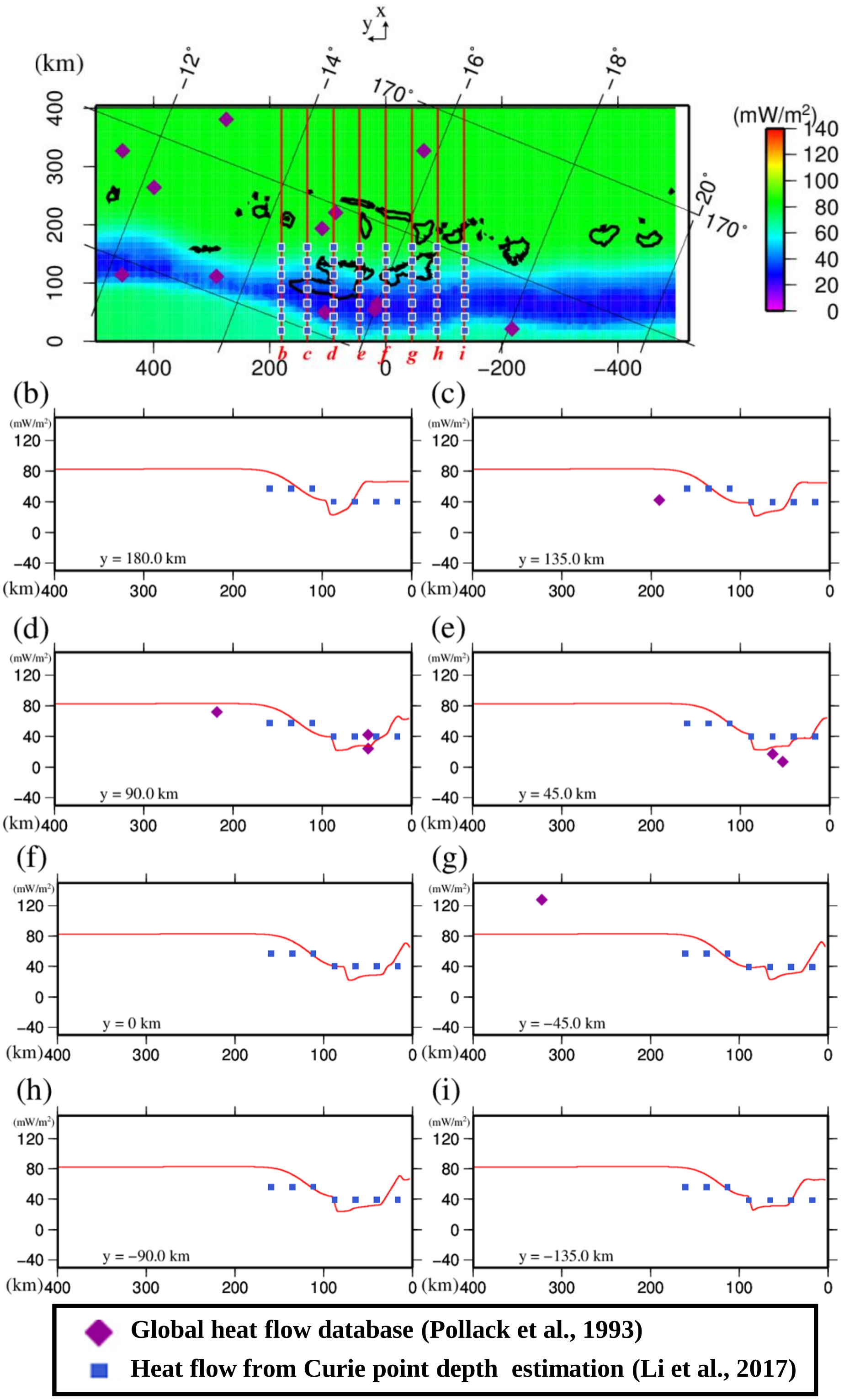

Fig. S1 (a) Spatial distribution of the surface heat flow calculated in this study. The observed and calculated surface heat flows are compared along trench-normal profiles “b” to “i” in panels (b) to (i). (b) Observed and calculated heat flows along profile b (y = 180 km) in (a). Purple diamonds

and blue squares denote heat flow data from the global heat flow database (Pollack et al., 1993) and heat flow from Curie point depth estimates (Li et al., 2017), respectively, within a width of 22.5 km along the profile. The red curve indicates the calculated heat flow along the profile. (c) Profile c (y = 135 km). (d) Profile d (y = 90 km). (e) Profile e (y = 45 km). (f) Profile f (y = 0 km). (g) Profile g (y = -45 km). (h) Profile h (y = -90 km). (i) Profile i (y = -135 km). The thermal conductivities K are assumed to be 1.5 W/m°C for the ocean (Grevemeyer et al., 2003; Li et al., 2017) and 2.5 W/m°C for the continent (Li et al., 2017).

Fig. S2

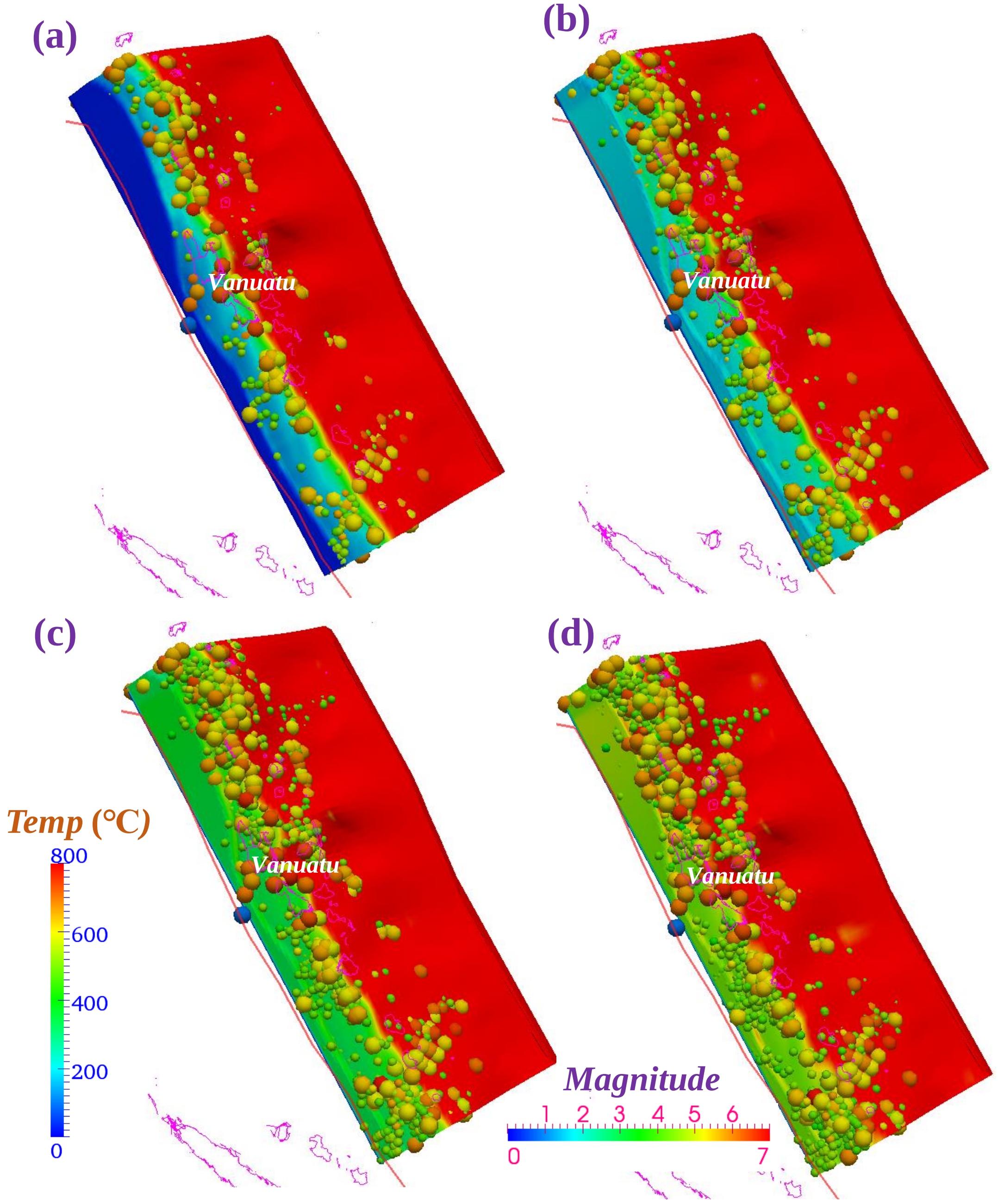

Fig. S2 Calculated thermal state of the incoming plate at various intraslab depths (measured vertically downward from the slab interface). The red cones indicate active arc volcanoes (Siebert et al., 2010). The colored spheres indicate the intraslab seismic events from January 2000 through December 2010 (IRIS; Trabant et al., 2012), with the color indicating the earthquake magnitude, corresponding to the "Magnitude" color scale at the bottom of the figure, and the size mimicking the rupture dimension. (a) The interface. (b) 8 km below the interface. (c) 16 km below the interface. (d) 24 km below the interface.

Fig. S3

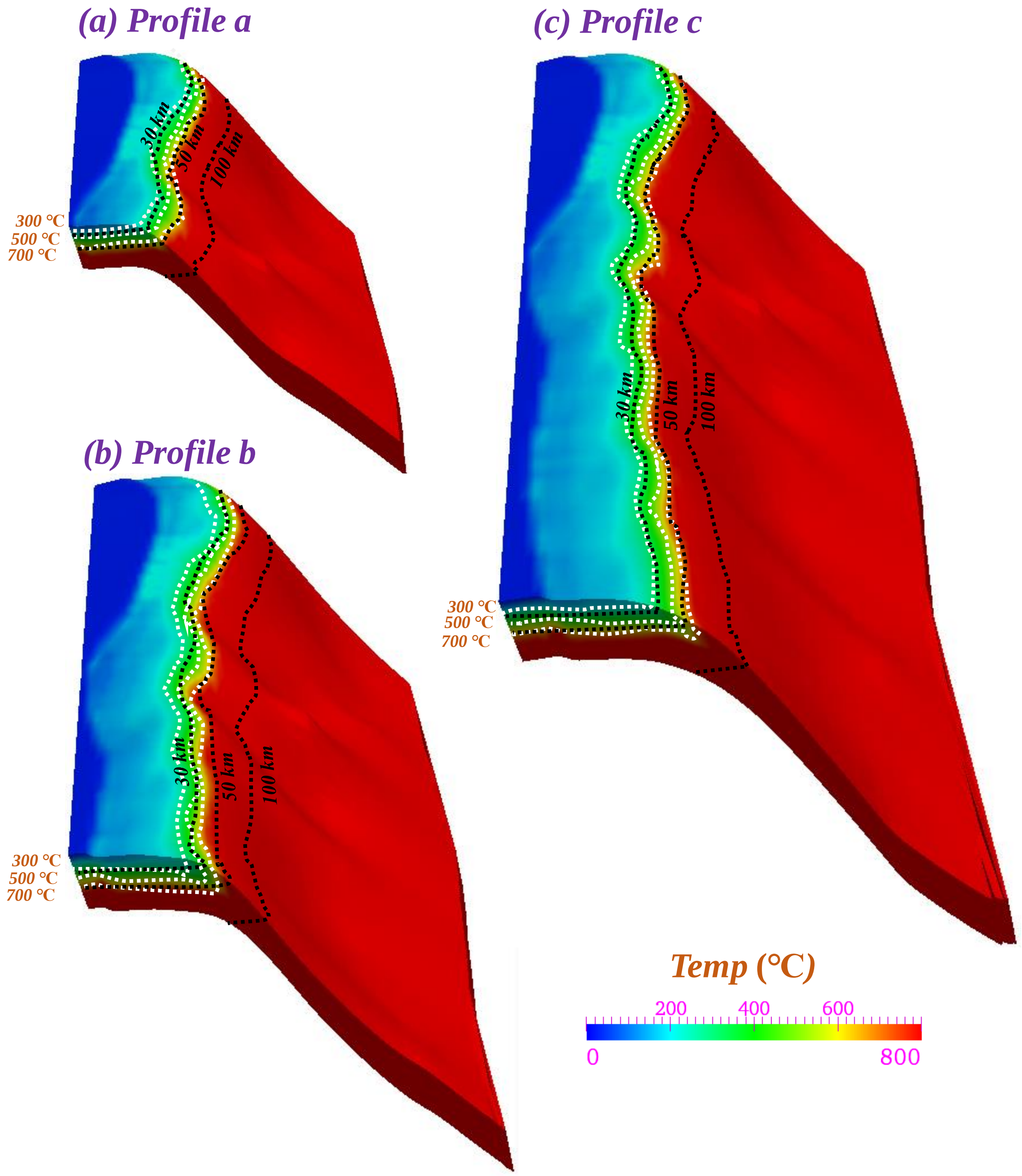

Fig. S3 Cross-sectional profiles of the temperature structure corresponding to profiles a, b, and c in Fig. 1. The white dashed lines represent the isotherm contours of 300°C, 500°C, and 700°C calculated by this study. The black dashed lines represent the depth contours of 30 km, 50 km, and 100 km.

Fig. S4

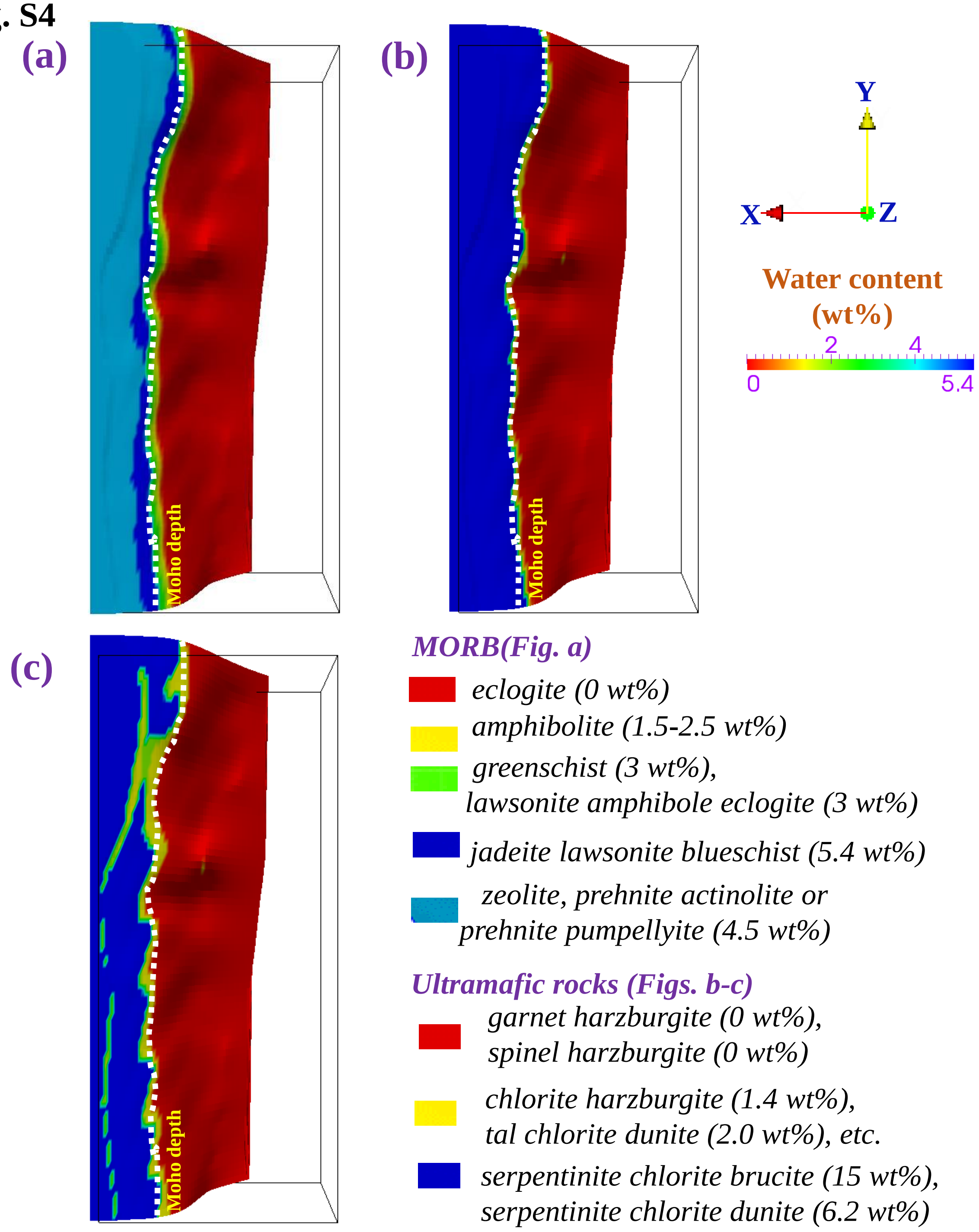

Fig. S4. Water content (wt%) of the incoming plate calculated by this study. The white dashed lines represent the depth contours of Moho depth. (a) The interface. (b) 16 km below the interface. (c) 24 km below the interface.

References

1. Argus, D. F., Gordon, R. G., DeMets, C., 2011. Geologically current motion of 56 plates relative to the no-net-rotation reference frame. Geochemistry, Geophysics, Geosystems 12(11).
2. Burkett, E.R., Billen, M.I., 2010. Three-dimensionality of slab detachment due to ridge-trench collision: Laterally simultaneous boudinage versus tear propagation, Geochemistry, Geophysics, Geosystems 11, Q11012, doi:10.1029/2010GC003286.
3. Christensen, U. R. 1996. The influence of trench migration on slab penetration into the lower mantle. Earth and Planetary Science Letters, 140(1-4), 27-39.
4. DeMets, C., Gordon, R.G., Argus, D.F., 2010. Geologically current plate motions, Geophysics. J. Int., 181, 1, 1-80, doi: 10.1111/j.1365-246X.2009.04491. x.
5. Grose, C.J., Afonso, J.C., 2013. Comprehensive plate models for the thermal evolution of oceanic lithosphere. Geochemistry, Geophysics, Geosystems 14, 3751-3778.
6. Hacker, B., Abers, G., Peacock, S., Holloway, S.D., 2003. Subduction factory 2. Are intermediate-depth earthquakes in subducting slabs linked to metamorphic dehydration reactions? Journal of Geophysical Research 108(B1):1-16.
7. Hayes, G.P., Moore, G.L., Portner, D.E., Hearne, M., Flamme, H., Furtney, M., Smoczyk, G.M., 2018. Slab2, a comprehensive subduction zone geometry model. Science 362, 58-61.
8. Hirth, G., Kohlstedt, D., 2003. Rheology of the upper mantle and the mantle wedge: A view from the experimentalists, in Inside the Subduction Factory, In J. Eiler (Ed.), Geophysical monograph series, Vol. 138, pp. 83–105, Washington, DC: AGU.
9. Iwamori, H., 1997. Heat sources and melting in subduction zones. J. Geophys. Res. Solid Earth 102, 14803-14820.
10. Ji, Y., Yoshioka, S., Banay, Y.A., 2017a. Thermal state, Slab metamorphism, and interface seismicity in the Cascadia subduction zone based on 3-D modeling. Geophysical research letters 44, 9242-9252.
11. Ji, Y., Yoshioka, S., Manea, V.C., Manea, M., 2017b. Seismogenesis of dual subduction beneath Kanto, central Japan controlled by fluid release. Scientific reports 7, 1-8.
12. Ji, Y., Yoshioka, S., Manea, V.C., Manea, M., Matsumoto, T., 2017c. Three-dimensional numerical modeling of thermal regime and slab dehydration beneath Kanto and Tohoku, Japan. Journal of Geophysical Research: Solid Earth 122, 332-353.
13. Ji, Y., Yoshioka, S., Matsumoto, T., 2016. Three-dimensional numerical modeling of temperature and mantle flow fields associated with subduction of the Philippine Sea plate, southwest Japan. Journal of Geophysical Research: Solid Earth 121(6), 4458-4482. doi:10.1002/2016JB012912.
14. Li, C.F., 2011. An integrated geodynamic model of the Nankai subduction zone and neighboring regions from geophysical inversion and modeling. J. Geodyn. 51, 64-80.
15. Li, C.F., Lu, Y., Wang, J., 2017. A global reference model of Curie-point depths based on EMAG2. Scientific reports 7, 1-9.
16. Li, Z.H., Xu, Z., Gerya, T., Burg, J.P., 2013. Collision of continental corner from 3-D numerical modeling. Earth and Planetary Science Letters, 380, 98-111.
17. Müller, R.D., Sdrolias, M., Gaina, C., Roest, W.R., 2008. Age, spreading rates, and spreading asymmetry of the world's ocean crust, Geochem. Geophysics, Geosystems 9 (4), Q04006, doi:10.1029/2007GC001743.
18. Omori, T., Watanabe, K., Umetsu, R. Y., Kainuma, R., Ishida, K., 2009. Martensitic transformation and magnetic field-induced strain in Fe-Mn-Ga shape memory alloy. Applied Physics Letters, 95(8), 082508.

19. Pollack, H.N., Hurter, S.J. Johnson, J.R. 1993. Heat flow from the earth's interior: analysis of the global data set. Reviews of Geophysics 31(3), 267-280.
20. Ranalli, G., 1995. Rheology of the earth. Tectonophysics 269(3), doi:10.1016/S0040-1951(96)00042-X.
21. Siebert, L., Simkin, T., Kimberly, P., 2010. Volcanoes of the World.
22. Stein, C. A., Stein, S., 1992. A model for the global variation in oceanic depth and heat flow with lithospheric age. Nature 359(6391), 123-129, doi:10.1038/359123a0.
23. Suenaga N., Yoshioka S., Ji, Y., 2021. 3-D thermal regime and dehydration processes around the regions of slow earthquakes along the Ryukyu Trench, Scientific Reports, 11(1), 11251, doi:10.1038/s41598-021-90199-2.
24. Tackley, P.J., Xie, S., 2003. Stag3D: A code for modeling thermochemical multiphase convection in Earth's mantle. Computational Fluid and Solid Mechanics 2003, edited by K. J. Bathe, pp. 1524-1527, Elsevier B.V., Amsterdam, Netherlands.
25. Trabant, C., Hutko, A. R., Bahavar, M., Karstens, R., Ahern, T., & Aster, R. (2012). Data products at the IRIS DMC: Stepping stones for research and other applications. Seismological Research Letters, 83(5), 846-854.
26. Turcotte, D.L., Schubert, G., 2002. Geodynamics, 2nd ed., pp. 186, Cambridge Univ. Press, Cambridge, U. K.
27. Wang, K., Hyndman, R.D., Yamano, M., 1995. Thermal regime of the Southwest Japan subduction zone: effects of age history of the subducting plate. Tectonophysics 248, 53-69.
28. White, R.S., McKenzie, D., O'Nions, R.K., 1992. Oceanic crustal thickness from seismic measurements and rare earth element inversions. Journal of Geophysical Research: Solid Earth 97, 19683-19715.
29. Yoshii, T., 1975. Regionality of group velocities of Rayleigh waves in the Pacific and thickening of the plate. Earth and Planetary Science Letters 25, 305-312.
30. Yoshioka, S., Murakami, K., 2007. Temperature distribution of the upper surface of the subducted Philippine Sea Plate along the Nankai Trough, southwest Japan, from a three-dimensional subduction model: relation to large interplate and low-frequency earthquakes. Geophysical Journal International.
